# Monocyte Phenotype and IFN-γ-Inducible Cytokine Responses Are Associated with Cryptococcal Immune Reconstitution Inflammatory Syndrome

**DOI:** 10.3390/jof3020028

**Published:** 2017-06-02

**Authors:** David B. Meya, Samuel Okurut, Godfrey Zziwa, Stephen Cose, Paul R. Bohjanen, Harriet Mayanja-Kizza, Moses Joloba, David R. Boulware, Carol Yukari Manabe, Sharon Wahl, Edward N. Janoff

**Affiliations:** 1 Infectious Diseases Institute, Makerere University, Kampala, P.O. Box 22418, Uganda; 2 Dept of Medicine, Center for Infectious Diseases and Microbiology Translational Research, University of Minnesota, Minneapolis, MN 55455, USA; bohja001@umn.edu (P.R.B.); boulw001@umn.edu (D.R.B.); 3 School of Medicine, College of Health Sciences, Makerere University, Kampala, P.O. Box 7072, Uganda; hmk@chs.mak.ac.ug; 4 Research Department, Makerere University Walter Reed Project, Plot 42, Nakasero Road, Kampala, P.O. Box 1624, Uganda; sokurut@muwrp.org (S.O.); gzziwa@muwrp.org (G.Z.); 5 London School of Hygiene & Tropical Medicine, Keppel Street, London WC1E 7HT, UK; stephen.cose@mrcuganda.org; 6 MRC/UVRI Uganda Research Unit on AIDS, UVRI, Entebbe, Plot 51-59 Nakiwogo Road, Uganda; 7 School of Biomedical Sciences, Microbiology Department, Makerere University, Kampala P.O. Box 7072, Uganda; m.joloba@gmail.com; 8 Division of Infectious Diseases, Department of Medicine, Johns Hopkins University, Baltimore, MD 21218, USA; ymanabe@jhmi.edu; 9 National Institute of Dental and Craniofacial Research, Bethesda, MD 20892, USA; SMWahl@dir.nidcr.nih.gov; 10 Mucosal and Vaccine Research Program Colorado (MAVRC), University of Colorado Denver, Aurora, CO 80045, USA; Edward.Janoff@ucdenver.edu

**Keywords:** cryptococcal meningitis, *Cryptococcus*, HIV, monocytes, innate immune response, IRIS

## Abstract

A third of adults with AIDS and cryptococcal meningitis (CM) develop immune reconstitution inflammatory syndrome (IRIS) after initiating antiretroviral therapy (ART), which is thought to result from exaggerated inflammatory antigen-specific T cell responses. The contribution of monocytes to the immunopathogenesis of cryptococcal IRIS remains unclear. We compared monocyte subset frequencies and immune responses in HIV-infected Ugandans at time of CM diagnosis (IRIS-Baseline) for those who later developed CM-IRIS, controls who did not develop CM-IRIS (Control-Baseline) at CM-IRIS (IRIS-Event), and for controls at a time point matched for ART duration (Control-Event) to understand the association of monocyte distribution and immune responses with cryptococcal IRIS. At baseline, stimulation with IFN-γ ex vivo induced a higher frequency of TNF-α- and IL-6-producing monocytes among those who later developed IRIS. Among participants who developed IRIS, ex vivo IFN-γ stimulation induced higher frequencies of activated monocytes, IL-6^+^, TNF-α^+^ classical, and IL-6^+^ intermediate monocytes compared with controls. In conclusion, we have demonstrated that monocyte subset phenotype and cytokine responses prior to ART are associated with and may be predictive of CM-IRIS. Larger studies to further delineate innate immunological responses and the efficacy of immunomodulatory therapies during cryptococcal IRIS are warranted.

## 1. Introduction

*Cryptococcus neoformans* is the most common cause of HIV-associated adult meningitis in sub-Saharan Africa, accounting for 15–20% of AIDS-related mortality [1,2]. The primary immune response to *Cryptococcus* involves fungal recognition by innate immune cells and subsequent expansion of *Cryptococcus*-specific CD4^+^ T cells to control the infection [3]. In immunocompromised persons, this immune sequence is impaired, resulting in ineffective fungal clearance. 

Up to 30% of patients with cryptococcal meningitis (CM) develop paradoxical immune reconstitution inflammatory syndrome (IRIS), which is a clinical deterioration after initiating antiretroviral therapy (ART) [4,5]. IRIS is proposed as an aberrant inflammatory response to persistent cryptococcal antigens during recovery of the host immune system [6]. These CM-IRIS events may manifest as relapsing aseptic meningitis, increased intracranial pressure, new focal neurologic signs, intracranial cryptococcomas, lymphadenopathy, and/or abscess formation [7,8,9]. 

CM-IRIS risk factors include higher fungal burden or cryptococcal antigen titer at CM diagnosis [9,10,11], positive cerebrospinal fluid (CSF) fungal culture when starting fluconazole 400 mg/day consolidation [12], and paucity of initial CSF immune response as suggested by low levels of: CSF white cells, interferon-γ (IFN-γ), tumor necrosis factor (TNF)-α, interleukin (IL)-2, IL-6, IL-8, and IL-17 [12,13,14].

The pathologic role of T cells in IRIS may derive from dysregulated expansion of activated antigen-specific T cells [15,16,17] resulting in inflammatory cytokine responses. However, neither the antigen-specificity of the T cell response during IRIS nor the role of innate myeloid cells in CM-IRIS has been well defined. The accumulation of primed antigen presenting cells is proposed to create a state of immunologic hyper-responsiveness to T-cell signaling upon immune recovery [9,10,11,12,13,14,15,16,17,18]. Thus, when T-cells are restored following ART initiation, antigen-specific CD4^+^ T-cells then provide a burst of IFN-γ with subsequent activation of the primed macrophages, which release proinflammatory cytokines, such as IL-6 and TNF-α. Innate cytokine signaling may precede IRIS-associated immunopathology [9].

In this study, we evaluated the role and function of peripheral blood monocytes from ART-treated HIV-infected participants treated for CM, and who did or did not subsequently develop CM-IRIS. By characterizing their phenotype and cytokine responses before and upon ex vivo stimulation with IFN-γ, a proxy for CD4^+^ T cell driven activation, we evaluated monocyte activation and functional patterns associated with CM-IRIS. 

## 2. Materials and Methods

### 2.1. Study Participants

We enrolled ART-naïve participants in a sub-study of the Cryptococcal Optimal ART Timing (COAT) Trial [19], a randomized trial testing the timing of early (1–2 weeks) versus deferred (4–6 weeks) ART initiation after cryptococcal meningitis. Participants received amphotericin-based (1 mg/kg) combination antifungal therapy with fluconazole (800 mg/day). ART consisted of zidovudine, lamivudine, and efavirenz. Peripheral blood mononuclear cells (PBMCs) were prospectively collected at serial longitudinal time points and at time of suspected possible IRIS event. A diagnosis of CM-IRIS was made per the consensus CM-IRIS case definition [20], with external adjudication by a three-physician panel and graded as definite, probable, or possible IRIS based on the available clinical and CSF information. Institutional review board approvals were obtained from the School of Medicine Ethics review committee at Makerere University (REF 2009-022, 29 January 2009) and the University of Minnesota (0810M49622, 11 February 2009), and written informed consent were obtained. 

### 2.2. Peripheral Blood Mononuclear Cells (PBMCs) Stimulation

PBMCs were isolated by density centrifugation gradient (Ficoll 1077, Sigma-Aldrich, St. Louis, MO, USA) and cryopreserved in RPMI-1640 with fetal bovine serum (20%), dimethyl sulphoxide (10%), and penicillin/streptomycin (1%), in liquid nitrogen. PBMCs (3–5) × 10^6^) were thawed in complete media (RPMI-1640 with 10% FBS, 2% HEPES, 2% l-Glutamine, 1% Pen/Strep) and incubated in triplicate wells with phosphate buffered saline (PBS; 100 μL/well) (Sigma-Aldrich, St. Louis, MO, USA) as negative control, lipopolysaccharide (LPS; 10 ng/mL) (Invivogen, San Diego, CA, USA) as positive control, or recombinant interferon-γ (IFN-γ; 150 U/mL) (Genentech, Inc., San Francisco, CA, USA).

### 2.3. Surface Flow Cytometric Staining

**Figure 1:**
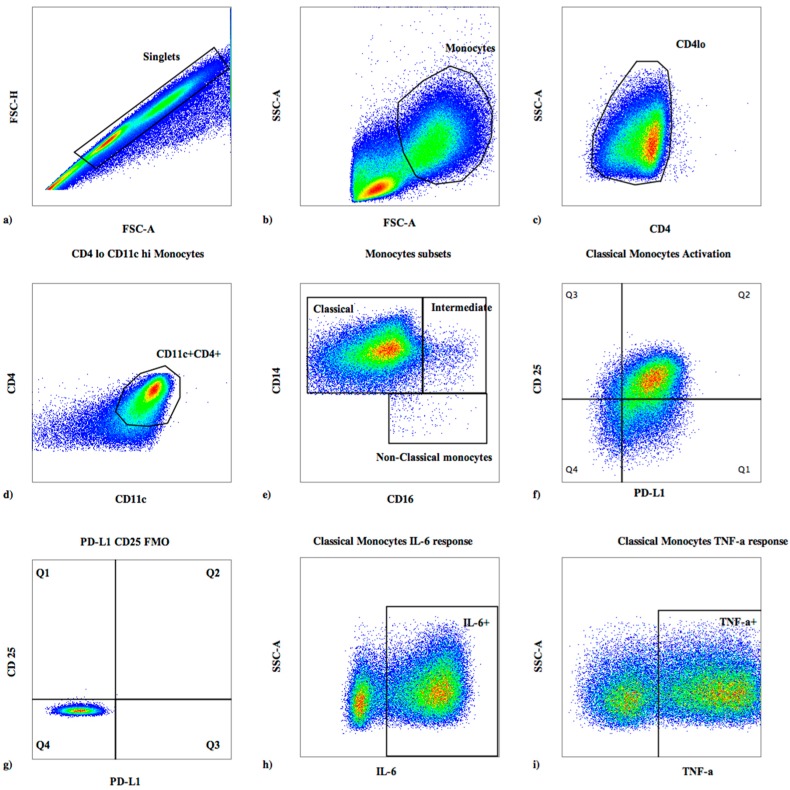
Flow cytometry gating strategy for monocytes. Analytic gating of flow cytometry data: (**a**) monocytes were identified from singlet cells; (**b**) monocytes were selected based on forward and side scatter; (**c**) cells expressing low levels of CD4 were selected; (**d**) cells co-expressing CD4^lo^CD11c were then identified; (**e**) gating on monocyte subsets was performed with classical monocytes as (CD14hi^++^CD16^−^), intermediate monocytes as (CD14^++^CD16^+^), and non-classical monocytes as (CD14^+^CD16^++^); (**f**) classical monocyte activation shown by co-expression of programmed death ligand-1 (PD-L1) and CD25 is shown; (**g**) gating on the fluorescence minus one (FMO) controls is used to set gates for CD25 and PD-L1; (**h**) expression of interleukin (IL)-6 by classical monocytes following interferon (IFN)-γ stimulation for a representative patient; (**i**) expression of tumor necrosis factor (TNF)-α by classical monocytes following IFN-γ stimulation for a representative patient.

Cell phenotype, activation state, and percentage of monocyte subsets were determined by 8-color flow cytometry using a FACSCanto II (BD Biosciences, San Jose, CA, USA). Differential gating was based on size and granularity (Figure 1a,b), with monocyte lineage defined as CD4^+lo^D11c^+^CD14^+^CD16^+/−^, classical monocytes as CD14^hi++^CD16^−^, intermediate monocytes as CD14^++^CD16^+^, and non-classical monocytes as CD14^+lo^CD16^++^ (Figure 1d) [21,22].

Cells were stained with monoclonal antibodies reactive with CD14^V500^ (clone M5E2), CD274/PD-L1^PE^ (clone MIH1), CD25^PE−Cy7^ (M-A251), CD16 ^APC−Cy7^ (clone 3G8), CD4^V450^ (clone RPA-T4) (BD Biosciences), and CD11c^PerCPCy5.5^ (clone 3.9, BioLegend, San Diego, CA, USA). Fluorescence minus one (FMO) controls were used to set gates for CD25 and programmed death-ligand 1 (PD-L1). Unstimulated samples were used to set gates for intracellular cytokines.

### 2.4. Intracellular Cytokine Staining

Monocyte cytokine responses were determined by intracellular cytokine staining. Following 2 h of stimulation at 37 °C, Brefeldin A (100 μL/mL) (BD) was added and cells were incubated for another 4 h, then overnight at 4 °C, and then washed, fixed, and permeabilized with successive washes in Fluorescence Activated Cell Sorter (FACS) permeabilizing solution (BD Biosciences). The cells were stained with surface antibodies to define phenotype as above and intracellularly with monoclonal antibodies reactive with IL-6^FITC^ (clone AS12, BD Horizon) and TNF-α^APC^ (clone MAb11, BioLegend). One million events were collected on a FACSCanto II and analyzed using FlowJo version 10.0.5 (TreeStar, Ashland, OR, USA).

### 2.5. Statistical Analysis

Data were analyzed using GraphPad Prism, version 6.0b (GraphPad Software Inc., La Jolla, CA, USA) and Spice, version 5.35 (NIAID, Bethesda, MD, USA). Paired samples at diagnosis and CM-IRIS were compared using the Wilcoxon signed-rank test. Cell phenotype and activation variables were compared between CM-IRIS and controls using the Mann-Whitney rank sum test. Significance was defined as *p*-value ≤ 0.05. Cytokine expression profiles were compared by permutation analysis, using a 10,000-iteration Monte Carlo simulation model, as described elsewhere [23].

## 3. Results

We enrolled 17 HIV-infected adults with CM, 11 of whom developed CM-IRIS and 6 of whom did not develop IRIS at Mulago Hospital in Kampala, Uganda between November 2010 and April 2012. Blood samples were obtained at CM-diagnosis (*n* = 15), IRIS-Baseline for participants who went on to develop CM-IRIS and Control-Baseline for controls who did not develop IRIS; at CM-IRIS (*n* = 11), (IRIS-Event), and controls without CM-IRIS at comparable ART duration (*n* = 6), (Control-Event).

Age, baseline CD4^+^ and CD8^+^ T cell counts, cryptococcal antigen (CrAg) titer, plasma HIV RNA (viral load), cerebrospinal fluid (CSF) protein, and white blood cells were similar among participants who later developed CM-IRIS and control participants (Table 1). At meningitis diagnosis, participants who later developed CM-IRIS showed a trend to higher median cryptococcal colony counts in CSF compared with those who did not develop IRIS (5.33 vs. 3.97 log10 colony forming units/mL (*p* = 0.078). Among the entire clinical cohort (*n* = 172), the median quantitative CSF culture was 5.1 log10 colony forming units/mL [19].

Among IRIS cases, the median duration from ART initiation to IRIS was 78 days (IQR, 43–144 days). The six control participants’ samples were obtained at diagnosis (Control-baseline) and at matched time points of ART duration (Control-event) with CM-IRIS subjects (±14 days), a median of 71 days (IQR, 63–84 days) after ART initiation.

At CM-IRIS (IRIS-event), the median serum CrAg titer had decreased three-fold from CM diagnosis (*p* = 0.078) (IRIS-baseline) (Table 1). Among controls, serum CrAg titer did not change significantly 81,920 (Interquartile range (IQR), 5,122–245,760) at CM diagnosis to 46,080 (IQR, 3–163,840; *p* = 0.681) at the later time point. CD4^+^ T cell numbers at baseline were low, particularly among those who developed IRIS (Table 1). By three months following initiation of ART, the proportion of CD4^+^ and CD8^+^ T cells increased in both populations (Table 1). However, at the time of CM-IRIS, the percent CD4^+^ T cells was lower among patients experiencing CM-IRIS (*p* = 0.039), respectively (Figure S1). In addition to smaller numbers of CD4^+^ T cells, we also considered that antigen presenting cell numbers and/or function might be contributing to CM-IRIS.

### 3.1. Baseline Monocyte Subsets

**Figure 2:**
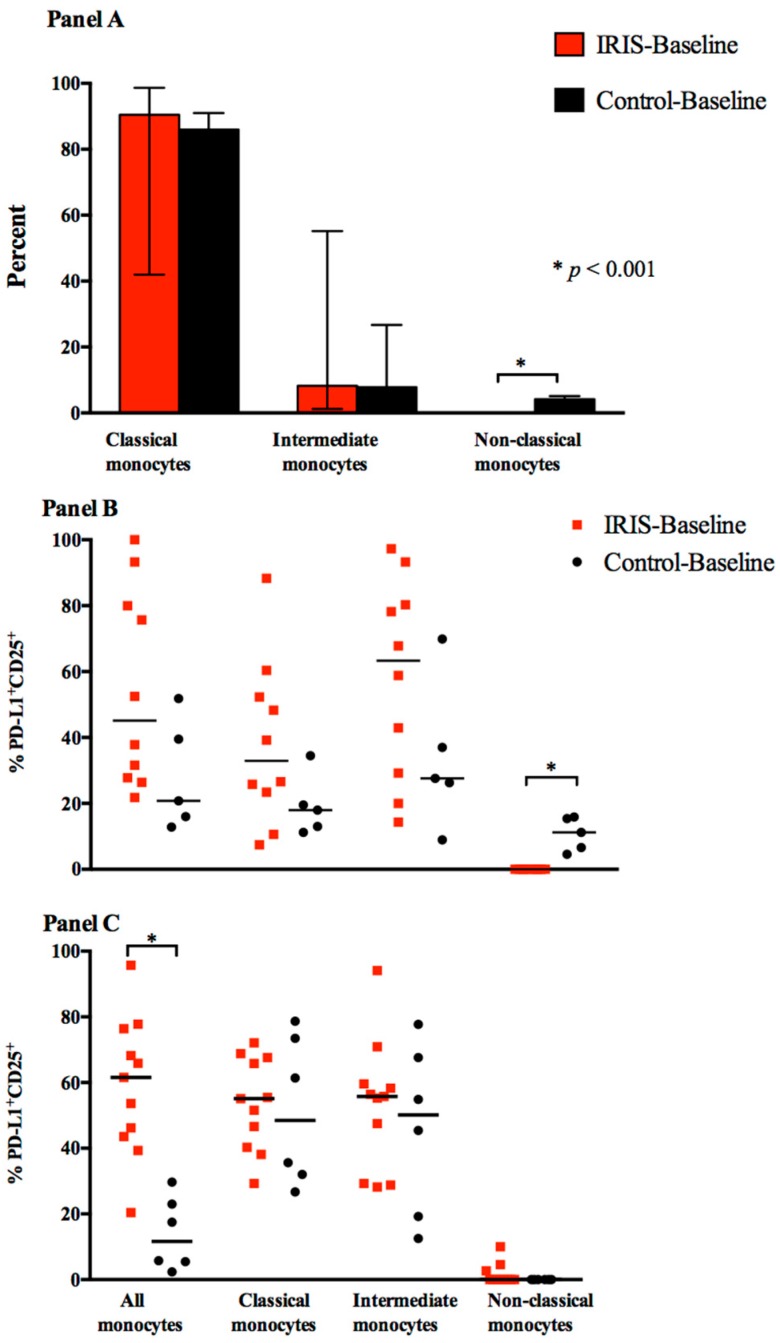
Monocyte Subsets and Activated (PD-L1^+^CD25^+^) Monocyte Subsets. (**Panel A**) displays total monocytes and monocyte subsets at CM diagnosis among participants who developed CM-IRIS (IRIS-baseline) vs. controls without IRIS (Control-baseline); (**Panel B**) displays activated monocytes and subsets at time of cryptococcal diagnosis from participants who subsequently developed CM-IRIS (IRIS-baseline) or matched controls on ART (Control-baseline); (**Panel C**) displays activated monocytes and subsets at IRIS-baseline and Control-baseline. Activation was measured by surface co-expression of programmed death ligand-1 (PD-L1) and CD25 (Interleukin 2 receptor α chain). Bars represent medians; * represents *p* < 0.001. Median values were compared using the Mann-Whitney test. Non-classical monocytes were absent at IRIS-baseline. Data is presented as percentage of monocytes but not percentage of total white blood cells, as the laboratory did not perform complete blood counts with every peripheral blood mononuclear cell (PBMC) isolation.

To evaluate a role for monocytic cells in the evolution of CM-IRIS, we measured the numbers of total monocytes and of the three phenotypic categories of monocyte subsets. Classical monocytes were the predominant subset at IRIS-Baseline and Control-Baseline (Figure 1d, Figure 2 panel A). Whereas no differences were detected in the intermediate subset, the frequencies of non-classical monocytes, the minority subset, were significantly lower at IRIS-Baseline vs. Control-Baseline (0% vs. 4.1%, *p* < 0.001) (Figure 2 panel A). The frequency of activated non-classical monocytes, as determined by co-expression of PD-L1 and CD25 (Figure 1e), was significantly lower in CM-IRIS compared with controls, 0% vs. 11% (6–16%), *p* < 0.001 (Figure 2 panel B). 

In contrast to baseline values, at the time of CM-IRIS, monocyte activation was significantly elevated in the total monocyte population from those with CM-IRIS compared with cells from time-matched controls, median frequency, (62% (IQR, 44–76%) vs. 12% (IQR, 5–25%); *p* < 0.001) (Figure 2 panel C), but these differences were not confined to one subset. These differences persisted from baseline to IRIS, and did not change significantly in either group. These data suggest that activation of monocytes both precedes and accompanies the development of CM-IRIS and may play a role in the immunopathogenesis of this clinical syndrome.

### 3.2. Cytokine Expression and Responses at Baseline

**Figure 3:**
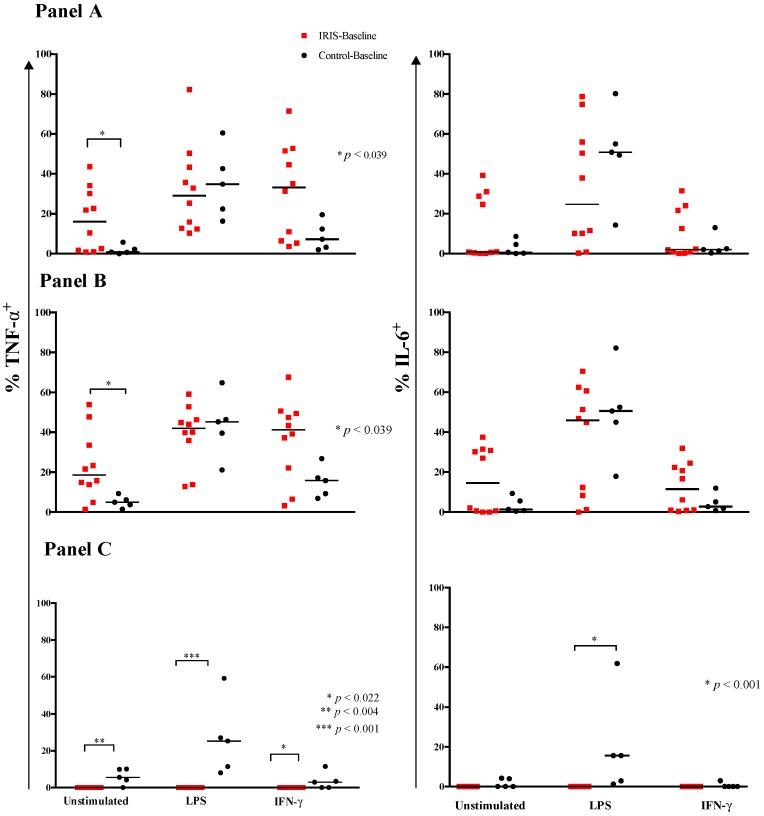
Cytokine Expression by monocytes at Cryptococcal Diagnosis. Frequencies of monocytes expressing intracellular TNF-α and IL-6 by flow cytometry at IRIS-Baseline and Control-Baseline. The frequencies of total monocytes expressing TNF-α or IL-6 (**Panel A**); classical monocytes expressing TNF-α or IL-6 (**Panel B**); and activated non-classical monocytes expressing TNF-α or IL-6 (**Panel C**) are shown. Cells were stimulated for 6 h with phosphate buffered saline (PBS), lipopolysaccharide (LPS; positive control), or the Th1 cytokine interferon-γ (IFN-γ; 150 U/mL). Horizontal bars represent the median value. Median values were compared using the Mann-Whitney test.

To characterize functional differences between monocyte populations isolated during IRIS-event and Control-event, we examined constitutive and induced expression of two relevant cytokines, TNF-α and IL-6. At IRIS-baseline, unstimulated monocytes consistently expressed a substantial and higher median frequency of TNF-α (16% (IQR, 2–31%) than Control-baseline, 0.8% (IQR, 0.3–4%); *p* = 0.039) (Figure 3 panel A). Results with IL-6 expression showed no differences. The TLR4 agonist, LPS, elicited a significant and comparable increase in expression of both cytokines in both groups (Figure 3 panel A), and significantly increased IL-6 expression compared to unstimulated monocytes among controls (Figure S2), highlighting the differences in cellular responses between the two groups. In contrast, stimulation with IFN-γ elicited increased expression of TNF-α and IL-6 by monocytes only at IRIS-Baseline. Indeed, following IFN-γ stimulation, the median frequency of activated monocytes expressing TNF-α or IL-6 was higher at IRIS-Baseline compared with Control-Baseline (Table 2). We found IFN-γ- induced IL-6 and TNF-α expression by activated (PD-L1^+^CD25^+^) monocytes was higher at IRIS-Baseline compared with Control-Baseline. Thus, spontaneous expression of TNF-α and, to a lesser extent, IL-6 were increased at IRIS-Baseline, as was the response of both cytokines to IFN-γ stimulation.

We determined the differential expression of these cytokines in the three monocyte subsets, including activated populations. Without stimulation, the frequency of TNF-α^+^ classical monocytes was higher at IRIS-Baseline compared with Control-Baseline (19% (IQR, 12–37%) vs. 5% (IQR, 3–8%); *p* = 0.039), whereas IL-6 expression did not differ (Figure 3 panel B). IL-6 expression by activated non-classical monocytes did not differ between the two groups, whereas TNF-α expression by the activated non-classical monocytes was lower at IRIS-Baseline compared with Control-Baseline, 0% vs. 6% (2–10%), *p* = 0.004 (Figure 3 panel C). 

Differential responses to IFN-γ among selective subsets were minimal. IFN-γ- inducible TNF-α^+^ and IL-6^+^ non-classical monocytes were less frequent at IRIS-Baseline (Table 2). Similarly, the proportion of IFN-γ activated (PD-L1^+^CD25^+^) TNF-α^+^ non-classical monocytes was nondetectable at IRIS-Baseline, compared to Control-Baseline, 0% vs. 3% (IQR, 0–7%) (*p* = 0.022).

### 3.3. Cytokine Responses at CM-IRIS

**Figure 4:**
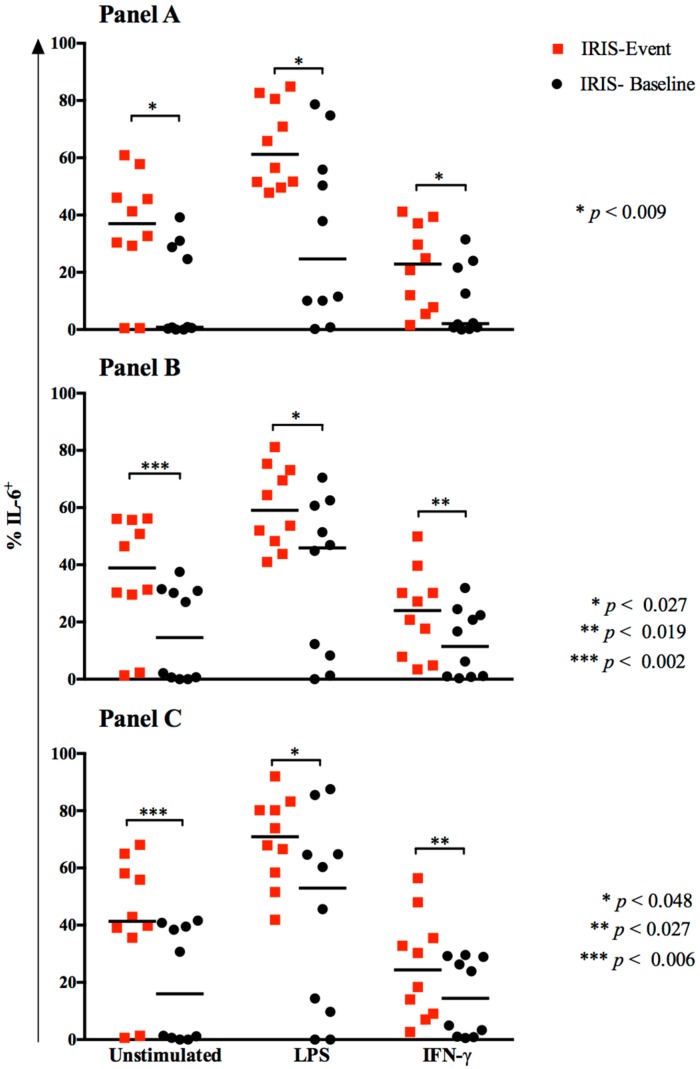
Comparison of IL-6 expression by monocyte populations at IRIS-Baseline vs. IRIS-Event among patients with paired samples. IL-6 intracellular expression was measured in total monocytes (**Panel A**); classical monocytes (**Panel B**), and activated (PDL1^+^CD25^+^) classical monocytes (**Panel C**) at IRIS-Baseline and IRIS-Event in 10 participants with paired samples. *p* values were determined by Wilcoxon rank sum test. At IRIS-Event, monocyte IL-6 expression was elevated compared to IRIS-Baseline from both unstimulated and IFN-γ-stimulated monocytes.

We next compared monocyte responses to IFN-γ in paired samples from the same participants at IRIS-Baseline and at IRIS-Event. The most prominent differences at IRIS-Event vs. IRIS-Baseline, respectively, were significantly increased frequencies of IL-6^+^ cells among total monocytes (23% (7–38%) vs. 1% (0.7–23%); *p* = 0.009), among classical monocytes, (24% (7–33%) vs. 11% (1–23%); *p* = 0.019) and among activated (CD25^+^PD-L1^+^) classical monocytes, (24% (9–39%) vs. 14% (1–29%), *p* = 0.027) (Figure 4) (data not shown). Thus, upregulation of IL-6 production was the most consistent intracellular cytokine marker of CM-IRIS.

Consistent with these paired results over time, the frequencies of IFN-γ-induced IL-6^+^ monocytes at IRIS-Event were also higher compared with Control-Event (Table 3). These differences extended to all monocyte subsets (Table 3, Figure S3). About a quarter to a third of each monocyte subset from IRIS-Event expressed IL-6 as well as TNF-α. Even in the absence of IFN-γ stimulation, the frequencies of TNF-α^+^ and IL-6^+^ monocytes were significantly elevated among IRIS-Event compared to Control-Event (Figure S3), further illustrating that monocyte activation and inflammatory mediators are linked to emergence of CM-IRIS. During IRIS-Event, constitutive and/or IFN-γ-induced cytokine responses were greater at IRIS-Event compared with Control-Event. A clear demonstration of IL-6 expression following different experimental conditions is shown in Figure S4. 

**Figure 5:**
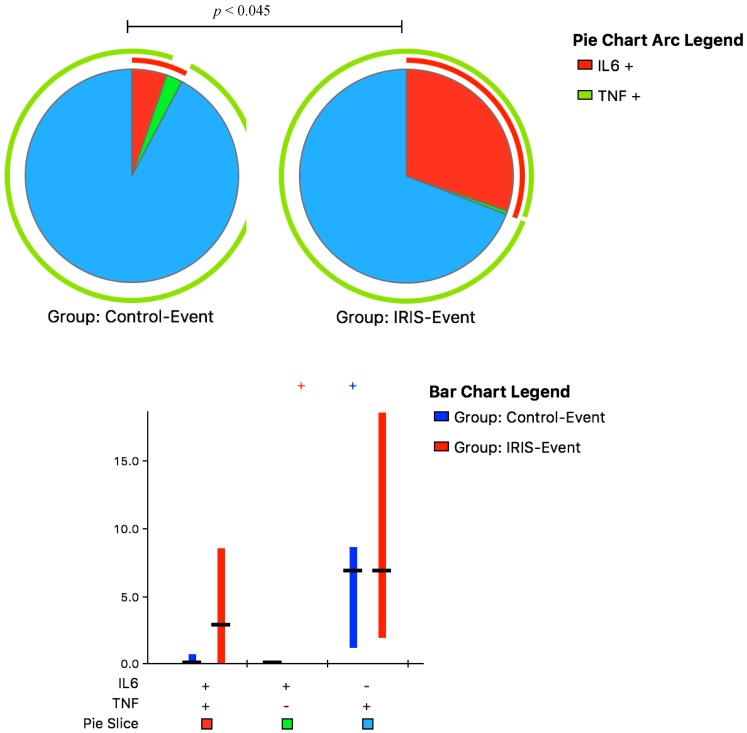
Functional Monocyte Cytokine responses to IFN-γ Stimulation. Peripheral blood mononuclear cells collected at IRIS-Event and Control-Event were stimulated with interferon-γ (IFN-γ). The bar chart shows each of the three possible combination responses on the *x*-axis. The percentage of the total cytokine response is shown on the *y*-axis, with the filled bar representing the interquartile range and a black line at the median. Responses at Control-Event are shown as blue bars, responses at IRIS-Event are shown as red bars on the graph. Statistically significant differences (*p* < 0.05) by rank-sum testing are indicated by the plus sign. The pie charts show the representative fractions according to the pie-slice colors shown at the bottom of the bar chart, with color-coded slices indicating the contributions of IL-6 and TNF-α (red), only IL-6 (green), and only TNF-α (blue) to the 2- and 1-function responses. Statistical comparisons of the overall responses by permutation testing are shown in the pie category test result chart where the red arc represents IL-6 expression and the green arc represents TNF-a producing cells. Patients with CM-IRIS had immune responses characterized by significantly elevated dual-function monocytes producing IL-6- and TNF-α compared to controls.

We assessed the monocyte functional phenotype following IFN-γ stimulation at IRIS-Event compared with Control-Event by measuring the possible combinations of 1- or 2-cytokine responses. At IRIS-Event, the immune responses were characterized by significantly elevated dual-function monocytes producing IL-6- and TNF-α, with a frequency of 39% (IQR, 20–61%) compared to 8% (IQR, 3–10%) at Control-Event, *p* = 0.045 (Figure 5). 

In summary, significantly different monocyte subset distribution, particularly non-classical monocytes, and function were detected at IRIS-Event and IRIS-Baseline compared to Control-Event and Control-Baseline. These differences extended to the distribution of monocyte subsets, particularly non-classical monocytes at baseline, to the degree of monocyte activation (CD25^+^PD-L1^+^) at IRIS-Event, and, in particular, to the expression of intracellular proinflammatory cytokines (IL-6 and, less consistently, TNF-α) prior to ART and subsequently at IRIS-Event with and without IFN-γ stimulation.

## 4. Discussion

There is a notable paucity of data on innate immune responses occurring during cryptococcal IRIS, and, in particular, the role of monocytes [24,25]. We have shown that monocyte phenotype and function may predict the incidence of cryptococcal IRIS, with the absence of non-classical monocytes at CM diagnosis and upregulation of IFN-γ induced IL-6 expression that precedes and accompanies the development of CM-IRIS.

Monocytes circulate in peripheral blood and migrate into tissues, maturing into phenotypically and functionally distinct macrophages at different anatomical locations. The main functions of monocytes include phagocytosis, antigen presentation, and cytokine production. The three subsets of circulating monocytes are characterized by the differential expression of surface CD14 and CD16: classical (CD14^hi++^CD16^−^), intermediate (CD14^++^CD16^+^), and non-classical monocytes (CD14^lo+^CD16^++^), each with certain unique phenotypic and functional parameters [21]. Classical monocytes can develop into intermediate monocytes and then to non-classical CD14^+^CD16^++^ monocytes, also described as patrolling monocytes [21]. The absence of non-classical monocytes prior to ART in patients with cryptococcal meningitis was associated with the incidence of IRIS, similar to paradoxical tuberculosis (TB)-IRIS, where the non-classical monocyte subset was also significantly decreased prior to ART initiation [26]. Non-classical monocytes have been demonstrated in CSF during the immune response to primary cryptococcal meningitis [25], however, it is unclear whether the absence of this monocyte subset was associated with poor cryptococcal antigen clearance with resulting CM-IRIS, as demonstrated in TB-IRIS [26]. 

During TB-IRIS, dysregulation of monocyte gene expression is functionally related to infection, inflammation, and inappropriate control of complement activation prior to initiating ART, during ART, and during the IRIS event [27,28]. Whether this scenario with an intracellular bacterial infection also occurs during the development of IRIS with an encapsulated fungal pathogen, such as *Cryptococcus*, is unknown. Barber and colleagues hypothesize that when patients with low CD4^+^ T cells acquire an opportunistic infection, subsequent paradoxical IRIS results from accumulated monocytes/macrophages harboring abundant organisms producing cytokines for cell signaling (e.g., IL-6), and then undergoing en masse activation upon an effective T cell response to produce excessive innate inflammatory cytokines. This activation coincides with the reconstitution of antigen-specific CD4^+^ T cells, which deliver previously diminished IFN-γ signaling as ART is initiated [18]. In this study, we provided a surrogate for restored T-cell signals to the monocyte population by in vitro stimulation of PBMCs with IFN-γ. By measuring the intracellular expression of the proinflammatory cytokines IL-6 and TNF-α in monocytes from patients with and without CM-IRIS, we simultaneously characterized measurement and phenotype of cytokine-producing monocytes at the single cell level [29].

We found elevated expression of TNF-α and IL-6 by monocytes at baseline among participants with future CM-IRIS, suggesting that elevation of these innate cytokines may predict future CM-IRIS, as demonstrated for IL-6 in TB or herpes virus-associated IRIS [30,31,32,33,34]. IL-6 induced during microbial infection influences macrophage differentiation and shifts the T helper 1(Th1)/Th2 balance toward the Th2 axis by promoting IL-4 production and Th2 differentiation to inhibit IFN-γ production and inhibition of regulatory T cells [9,35]. Another important pathway in the immune response against fungal infections is Th17, which is associated with increased IFN-γ production by T cells and influenced by IL-6 kinetics [36]. *Cryptococcus* similarly promotes an imbalance toward a Th2 response by suppressing Th1 responses via IL-12 produced by macrophages [37,38,39]. Together, the influence of IL-6 and cryptococcal infection could promote poor antigen clearance, persistence of residual antigen, and subsequent risk of CM-IRIS, consistent with the elevated serum CrAg titer among those pre-disposed to CM-IRIS.

We found elevated monocyte IL-6 expression among participants with CM-IRIS compared to controls without IRIS. Barber and colleagues have demonstrated in a murine model of experimentally-inducible *Mycobacterium avium* IRIS that by neutralizing IL-6, C-reactive protein levels diminished, wasting disease was alleviated, and host survival was prolonged [40]. Further, combined blockade of IL-6 and IFN-γ further reduced IRIS pathology even after onset of wasting disease, pointing to the possible role of the IL-6 pathway in the immunopathology of IRIS. Our results are consistent with a few other human studies that have demonstrated elevated IL-6 during CM-IRIS, which show different cytokine responses in CSF and blood [9,13]. We posit that an anti-IL-6 monoclonal antibody (e.g., siltuximab) could be further evaluated in understanding the efficacy of alleviating the pathology and mortality associated with cryptococcal IRIS in the enclosed central nervous system space cognizant of its pleotropic nature. Despite the increased risk of developing cryptococcosis among patients using anti-TNF antagonists [41,42], a therapeutic option to evaluate in larger studies would be to target inhibition of TNF-α for the alleviation of the neurological symptoms associated with CM-IRIS, as suggested in two case reports [43,44]. However, the clinical response may be variable by person.

The frequency of activated monocytes co-expressing PD-L1 and CD25 was increased at CM-IRIS compared with that in matched controls. PD-L1, expressed by various effector cytokines, has the ability to co-stimulate naïve T cells and co-inhibit effector T cells through the programmed cell death protein 1 (PD-1) receptor, and is expressed on most activated immune cells [45,46]. IFN-γ induces monocytes to express TNF-α and IL-6, both of which were elevated during CM-IRIS compared to CM diagnosis and to controls without CM-IRIS in response to IFN-γ stimulation. Thus, increased monocyte activation and restored cytokine responses to IFN-γ signaling from recovering CD4^+^ T cells appear to coincide with the timing of CM-IRIS, consistent with the prior proposed paradigm [9,18]. 

Taken together, elevated frequencies of activated monocytes, our previously published data showing the presence of proinflammatory intermediate monocytes in CSF [47], the elevation of IL-6 during CM-IRIS [9,13], and our data showing increased monocyte cytokines at baseline suggest monocyte dysregulation associated with augmented disease. Moreover, data showing increased CCL2 and CCL3 chemokines in the CSF compared with blood [48], and the association between baseline CCL2, CCL3, and GM-CSF expression in CSF and subsequent IRIS [49], suggest enhanced trafficking of activated monocytes from blood into the CSF during CM-IRIS. In addition, within the overall COAT trial, we found that earlier ART initiation was associated with CSF microglial/macrophage activation and increased cellular infiltrate in persons with cryptococcal meningitis [50].

Higher fungal burden or cryptococcal antigen titer at CM diagnosis is known to be a risk factor for poor outcomes and CM-IRIS [9,10,11]. Participants with CM-IRIS in our study had a tendency to have higher cryptococcal quantitative colony counts compared to control participants at baseline. 

Despite the small sample size, our findings provide a basis for further examining correlations between monocyte subsets, antigen burden, cytokine responses, and clinical outcomes in larger studies. We did not have a sufficient sample size to evaluate the correlation of our findings with ART timing, as there were only six participants with IRIS in the deferred arm and four participants in the early arm of the COAT trial [19]. We studied monocyte function in peripheral blood and not directly in the CNS, the site of CM-IRIS pathology, thus limiting extrapolation to CSF. Although these data may not mirror compartment-specific responses, they may be reflective of monocytes already exposed and exhausted following IFN-γ signaling in vivo during CM-IRIS, thus we interpret the cytokine responses with some caution. Due to the overlap and a degree of heterogeneity in IL-6 responses among those who developed CM-IRIS and controls who did not develop CM-IRIS, we can only surmise that IL-6 could be a co-predictor of increased risk for CM- IRIS. Nevertheless, building on our previous studies of CM-IRIS using cytokine levels in serum [9] and CSF [13], an instructive feature of this study is the detection of augmented monocyte cytokine responses at the single cell level prior to the development of and during cryptococcal IRIS. There was likely a gender bias in the selection of controls, since we did not match on gender, however, we do not envisage any correlation between gender and cytokine expression during IRIS.

In conclusion, we have demonstrated that monocyte subset phenotype and cytokine responses prior to ART are associated with and may be predictive of CM-IRIS. Larger studies to further delineate innate immunological responses and the efficacy of immunomodulatory therapies during cryptococcal IRIS are warranted. 

## Figures and Tables

**Table 1:** Characteristics of Participants Who Developed Cryptococcal Meningitis (CM)-immune reconstitution inflammatory syndrome (IRIS) vs. Participants without CM-IRIS.

Variable	Controls (*n* = 6)	CM-IRIS (*n* = 11)	*p*-Value
Male %	17%	73%	0.05
Age years	35 (±9)	35 (±8)	0.937
Baseline CD4 T cells/µL	8 (5, 166)	6.5 (4, 28)	0.828
CD4 T cells/µL > 3 months after ART	156 (55, 309)	68 (33, 79)	0.256
Baseline CD8 T cells/µL	163 (97, 784)	256 (140, 591)	0.515
CD8 T cells/µL > 3 months after ART	1005 (615, 1086)	831 (565, 997)	0.463
Baseline plasma HIV-1 RNA (log10copies/mL)	4.93 (±0.34)	5.24 (±0.5)	0.280
Baseline *Cryptococcus* log10 colony forming units/mL	3.97 (2.45, 5.26)	5.33 (4.96, 5.46)	0.078
Baseline CSF CrAg titer	4512 (528, 12,192)	7,200 (4048, 16,384)	0.455
Serum CrAg titer at CM-IRIS		40,960 (8960, 102,400)	-
CSF CrAg titer at CM-IRIS		12,000 (4672, 16,384)	-
CSF protein (mg/dL)	60 (47, 68)	53 (20, 70)	0.471
Baseline CSF white cells/mL	19.5 (<5, 45)	<5 (<5, <5)	0.169
Duration from ART initiation (days)	67 (48, 92)	78 (43, 202)	0.737

All values listed as median (IQR) or mean (± SD). Abbreviations: CrAg—Cryptococcal Antigen; CSF—Cerebrospinal Fluid; ART—Antiretroviral Therapy; HIV—Human Immunodeficiency Virus. Comparison was done using a *t*-test.

**Table 2:** Cytokine expression in circulating monocytes after stimulation with interferon-γ at control-baseline and IRIS-Baseline.

Monocyte Population with Intracellular Cytokine Production	Control-Baseline (*n* = 5)	IRIS-Baseline (*n* = 10)	*p*-Value
All Monocytes			
TNF-α^+^	7 (3, 16)	33 (6, 52)	0.096
IL-6^+^	2.0 (0.8, 7.7)	2.0 (0.6, 22)	0.893
TNF-α^+^ or IL-6^+^	8 (3, 18)	35 (6, 53)	0.050
Activated Monocytes (PD-L1^+^CD25^+^)			
TNF-α^+^	11 (4, 21)	50 (23, 55)	0.026
IL-6^+^	3 (1, 13)	27 (6, 28)	0.018
Activated Classical Monocytes (PD-L1^+^CD25^+^)			
TNF-α^+^	13 (7, 26)	42 (19, 56)	0.073
IL-6^+^	3 (1, 13)	14 (1, 29)	0.196
Intermediate Monocytes			
TNF-α^+^	9 (5, 31)	29 (9, 52)	0.291
IL-6^+^	5 (2, 36)	13 (1, 33)	0.834
Activated Intermediate Monocytes (PD-L1^+^CD25^+^)			
TNF-α^+^	9 (6, 28)	36 (8, 69)	0.240
IL-6^+^	5 (1, 41)	18 (2, 44)	0.743
Activated Non-Classical monocytes (PD-L1^+^CD25^+^)			
TNF-α^+^	3.0 (0, 7)	0.0	0.022
IL-6^+^	0.0 (0, 2)	0.0	0.333

All values listed as median % (IQR) unless otherwise noted. *p*-value determined from Mann-Whitney U test. Abbreviations: TNF—tumor necrosis factor; IL—Interleukin. Classical monocytes defined as (CD14^hi++^CD16^−^), intermediate monocytes as (CD14^++^CD16^+^), and non-classical monocytes as (CD14^+lo^CD16^++^). One control subject did not have PBMCs collected at CM diagnosis.

**Table 3:** Cytokine Expression in Circulating Monocytes after Stimulation with Interferon-γ at IRIS-Event or Control-Event.

Monocyte Population with Intracellular Cytokine Production	Control-Event (*n* = 6)	IRIS-Event (*n* = 11)	*p*-Value
All Monocytes			
TNF-α^+^	8 (2, 10)	22 (7, 52)	0.091
IL-6^+^	0.6 (0.4, 1.3)	25 (8, 37)	<0.001
TNF-α^+^ and IL-6^+^	8 (3, 10)	39 (20, 61)	0.003
Activated Monocytes (PD-L1^+^CD25^+^)			
TNF-α^+^	10 (6, 18)	36 (24, 64)	0.007
IL-6^+^	0.4 (0.2, 1.0)	36 (7, 44)	<0.001
Classical Monocytes			
TNF-α^+^	9 (3, 14)	29 (23, 47)	0.005
IL-6^+^	1.5 (0.4, 2.0)	24 (8, 30)	<0.001
Activated Classical Monocytes			
TNF-α^+^	7 (1, 17)	26 (17, 45)	0.010
IL-6^+^	0.6 (0.4, 1.3)	21 (9, 36)	<0.001
Intermediate Monocytes			
TNF-α^+^	7 (4, 11)	25 (16, 76)	0.020
IL-6^+^	0.9 (0.4, 1.5)	23 (7, 40)	<0.001
Activated Intermediate Monocytes			
TNF-α^+^	6 (5, 13)	24 (13, 72)	0.025
IL-6^+^	0.5 (0.1, 0.7)	25 (8, 46)	0.003
Non-Classical Monocytes			
TNF-α^+^	0	0 (0, 39)	0.102
L-6^+^	0	0 (0, 16)	0.102
Activated Non-Classical Monocytes			
TNF-α^+^	0	0 (0, 80)	0.353
IL-6^+^	0	0	0.515

Control-Event (*n* = 6) participants were matched for ART duration to the 11 participants at IRIS-Event. All values listed as median % (IQR) unless otherwise noted. *p*-value determined from Mann-Whitney U test. Abbreviations: TNF—tumor necrosis factor; IL—Interleukin. Classical monocytes defined as (CD14^hi++^CD16^−^), intermediate monocytes as (CD14^++^CD16^+^), and non-classical monocytes as (CD14^+lo^CD16^++^).

## Supplementary Materials

Supplementary materials can be found at: www.mdpi.com/2309-608X/3/2/28/s1.

## References

[1] French N., Gray K., Watera C., Nakiyingi J., Lugada E., Moore M., Lalloo D., Whitworth J.A., Gilks C.F. (2002). Cryptococcal infection in a cohort of HIV-1-infected Ugandan adults. AIDS.

[2] Hakim J.G., Gangaidzo I.T., Heyderman R.S., Mielke J., Mushangi E., Taziwa A., Robertson V.J., Musvaire P., Mason P.R., Hakim J.G. (2000). Impact of HIV infection on meningitis in Harare, Zimbabwe: A prospective study of 406 predominantly adult patients. AIDS.

[3] Huffnagle G.B., Lipscomb M.F. (1998). Cells and cytokines in pulmonary cryptococcosis. Res. Immunol..

[4] Lortholary O., Fontanet A., Mémain N., Martin A., Sitbon K., Dromer F., French Cryptococcosis Study Group (2005). Incidence and risk factors of immune reconstitution inflammatory syndrome complicating HIV-associated cryptococcosis in France. AIDS.

[5] Muller M., Wandel S., Colebunders R., Attia S., Furrer H., Egger M., for IeDEA Southern and Central Africa (2010). Immune reconstitution inflammatory syndrome in patients starting antiretroviral therapy for HIV infection: A systematic review and meta-analysis. Lancet Infect. Dis..

[6] French M.A. (2009). HIV/AIDS: Immune reconstitution inflammatory syndrome: A reappraisal. Clin. Infect. Dis..

[7] Cattelan A.M., Trevenzoli M., Sasset L., Lanzafame M., Marchioro U., Meneghetti F. (2004). Multiple cerebral cryptococcomas associated with immune reconstitution in HIV-1 infection. AIDS.

[8] Boelaert J.R., Goddeeris K.H., Vanopdenbosch L.J., Casselman J.W. (2004). Relapsing meningitis caused by persistent cryptococcal antigens and immune reconstitution after the initiation of highly active antiretroviral therapy. AIDS.

[9] Boulware D.R., Meya D.B., Bergemann T.L., Wiesner D.L., Abdu Musubire J.R., Lee S.J., Kambugu A., Janoff E.N., Bohjanen P.R. (2010). Clinical features and serum biomarkers in HIV immune reconstitution inflammatory syndrome after cryptococcal meningitis: A prospective cohort study. PLoS Med..

[10] Sungkanuparph S., Filler S.G., Chetchotisakd P., Pappas P.G., Nolen T.L., Manosuthi W., Anekthananon T., Morris M.I., Supparatpinyo K., Kopetskie H. (2009). Cryptococcal immune reconstitution inflammatory syndrome after antiretroviral therapy in AIDS patients with cryptococcal meningitis: A prospective multicenter study. Clin. Infect. Dis..

[11] Shelburne S.A., Darcourt J., White A.C., Greenberg S.B., Hamill R.J., Atmar R.L., Visnegarwala F. (2005). The role of immune reconstitution inflammatory syndrome in AIDS-related Cryptococcus neoformans disease in the era of highly active antiretroviral therapy. Clin. Infect. Dis..

[12] Chang C.C., Dorasamy A.A., Gosnell B.I., Elliott J.H., Spelman T., Omarjee S., Naranbhai V., Coovadia Y., Ndung’u T., Moosa M.Y. (2013). Clinical and mycological predictors of cryptococcosis-associated immune reconstitution inflammatory syndrome. AIDS.

[13] Boulware D.R., Bonham S.C., Meya D.B., Wiesner D.L., Park G.S., Kambugu A., Janoff E.N., Bohjanen P.R. (2010). Paucity of initial cerebrospinal fluid inflammation in cryptococcal meningitis is associated with subsequent immune reconstitution inflammatory syndrome. J. Infect. Dis..

[14] Jarvis J.N., Meintjes G., Rebe K., Williams G.N., Bicanic T., Williams A., Schutz C., Bekker L.G., Wood R., Harrison T.S. (2012). Adjunctive interferon-gamma immunotherapy for the treatment of HIV-associated cryptococcal meningitis: A randomized controlled trial. AIDS.

[15] Antonelli L.R., Mahnke Y., Hodge J.N., Porter B.O., Barber D.L., DerSimonian R., Greenwald J.H., Roby G., Mican J., Sher A. (2010). Elevated frequencies of highly activated CD4+ T cells in HIV+ patients developing immune reconstitution inflammatory syndrome. Blood.

[16] Mahnke Y.D., Greenwald J.H., DerSimonian R., Roby G., Antonelli L.R., Sher A., Roederer M., Sereti I. (2012). Selective expansion of polyfunctional pathogen-specific CD4(+) T cells in HIV-1-infected patients with immune reconstitution inflammatory syndrome. Blood.

[17] Bourgarit A., Carcelain G., Martinez V., Lascoux C., Delcey V., Gicquel B., Vicaut E., Lagrange P., Sereni D., Autran B. (2006). Explosion of tuberculin-specific Th1-responses induces immune restoration syndrome in tuberculosis and HIV co-infected patients. AIDS.

[18] Barber D.L., Andrade B.B., Sereti I., Sher A. (2012). Immune reconstitution inflammatory syndrome: The trouble with immunity when you had none. Nat. Rev. Microbiol..

[19] Boulware D.R., Meya D.B., Muzoora C., Rolfes M.A., Hullsiek K.H, Musubire A., Taseera K., Nabeta H.W., Schutz C., Williams D.A. (2014). Timing of antiretroviral therapy after diagnosis of cryptococcal meningitis. N. Engl. J. Med..

[20] Haddow L.J., Colebunders R., Meintjes G., Lawn S.D., Elliott J.H., Manabe Y.C., Bohjanen P.R., Sungkanuparph S., Easterbrook P.J., French M.A. (2010). Cryptococcal immune reconstitution inflammatory syndrome in HIV-1-infected individuals: Proposed clinical case definitions. Lancet Infect. Dis..

[21] Ziegler-Heitbrock L. (2007). The CD14+ CD16+ blood monocytes: Their role in infection and inflammation. J. Leukoc Biol..

[22] Ziegler-Heitbrock L., Ancuta P., Crowe S., Dalod M., Grau V., Hart D.N., Leenen P.J., Liu Y.J., MacPherson G., Randolph G.J. (2010). Nomenclature of monocytes and dendritic cells in blood. Blood.

[23] Roederer M., Nozzi J.L., Nason M.C. (2011). SPICE: Exploration and analysis of post-cytometric complex multivariate datasets. Cytom. A.

[24] Chretien F., Lortholary O., Kansau I., Neuville S., Gray F., Dromer F. (2002). Pathogenesis of cerebral Cryptococcus neoformans infection after fungemia. J. Infect. Dis..

[25] Naranbhai V., Chang C.C., Durgiah R., Omarjee S., Lim A., Moosa M.Y., Elliot J.H., Ndung’u T., Lewin S.R., French M.A. (2014). Compartmentalization of innate immune responses in the central nervous system during cryptococcal meningitis/HIV coinfection. AIDS.

[26] Andrade B.B., Singh A., Narendran G., Schechter M.E., Nayak K., Subramanian S., Anbalagan S., Jensen S.M., Porter B.O., Antonelli L.R. (2014). Mycobacterial Antigen Driven Activation of CD14++CD16− Monocytes Is a Predictor of Tuberculosis-Associated Immune Reconstitution Inflammatory Syndrome. PLoS Pathog..

[27] Tran H.T., Van den Bergh R., Vu T.N., Laukens K., Worodria W., Loembe M.M., Colebunders R., Kestens L., De Baetselier P., Raes G. (2014). The role of monocytes in the development of Tuberculosis-associated Immune Reconstitution Inflammatory Syndrome. Immunobiology..

[28] Tran H.T., Van den Bergh R., Loembe M.M., Worodria W., Mayanja-Kizza H., Colebunders R., Mascart F., Stordeur P., Kestens L., De Baetselier P. (2013). Modulation of the complement system in monocytes contributes to tuberculosis-associated immune reconstitution inflammatory syndrome. AIDS.

[29] Gauduin M.C., Kaur A., Ahmad S., Yilma T., Lifson J.D., Johnson R.P. (2004). Optimization of intracellular cytokine staining for the quantitation of antigen-specific CD4+ T cell responses in rhesus macaques. J. Immunol. Methods.

[30] Porter B.O., Ouedraogo G.L., Hodge J.N., Smith M.A., Pau A., Roby G., Kwan R., Bishop R.J., Rehm C., Mican J. (2010). d-Dimer and CRP levels are elevated prior to antiretroviral treatment in patients who develop IRIS. Clin. Immunol..

[31] Rodger A.J., Fox Z., Lundgren J.D., Kuller L.H., Boesecke C., Gey D., Skoutelis A., Goetz M.B., Phillips A.N., Group ISfMoATS (2009). Activation and coagulation biomarkers are independent predictors of the development of opportunistic disease in patients with HIV infection. J. Infect. Dis..

[32] Kalayjian R.C., Machekano R.N., Rizk N., Robbins G.K., Gandhi R.T., Rodriguez B.A., Pollard R.B., Lederman M.M., Landay A. (2010). Pretreatment levels of soluble cellular receptors and interleukin-6 are associated with HIV disease progression in subjects treated with highly active antiretroviral therapy. J. Infect. Dis..

[33] Stone S.F., Price P., Brochier J., French M.A. (2001). Plasma bioavailable interleukin-6 is elevated in human immunodeficiency virus-infected patients who experience herpesvirus-associated immune restoration disease after start of highly active antiretroviral therapy. J. Infect. Dis..

[34] Stone S., Price P., Keane N., Murray R., French M. (2002). Levels of IL-6 and soluble IL-6 receptor are increased in HIV patients with a history of immune restoration disease after HAART. HIV Med..

[35] Diehl S., Rincon M. (2002). The two faces of IL-6 on Th1/Th2 differentiation. Mol. Immunol..

[36] Murdock B.J., Huffnagle G.B., Olszewski M.A., Osterholzer J.J. (2014). Interleukin-17A enhances host defense against cryptococcal lung infection through effects mediated by leukocyte recruitment, activation, and gamma interferon production. Infect. Immun..

[37] Koguchi Y., Kawakami K. (2002). Cryptococcal infection and Th1-Th2 cytokine balance. Int. Rev. Immunol..

[38] Vecchiarelli A., Retini C., Pietrella D., Monari C., Tascini C., Beccari T., Kozel T.R. (1995). Downregulation by cryptococcal polysaccharide of tumor necrosis factor alpha and interleukin-1 beta secretion from human monocytes. Infect. Immun..

[39] Chaka W., Heyderman R., Gangaidzo I., Robertson V., Mason P., Verhoef J., Verheul A., Hoepelman A.I. (1997). Cytokine profiles in cerebrospinal fluid of human immunodeficiency virus-infected patients with cryptococcal meningitis: No leukocytosis despite high interleukin-8 levels. University of Zimbabwe Meningitis Group. J. Infect. Dis..

[40] Barber D.L., Andrade B.B., McBerry C., Sereti I., Sher A. (2014). Role of IL-6 in Mycobacterium avium—Associated immune reconstitution inflammatory syndrome. J. Immunol..

[41] Wilson M.L., Sewell L.D., Mowad C.M. (2008). Primary cutaneous Cryptococcosis during therapy with methotrexate and adalimumab. J. Drugs Dermatol..

[42] Salmon-Ceron D., Tubach F., Lortholary O., Chosidow O., Bretagne S., Nicolas N., Cuillerier E., Fautrel B., Michelet C., Morel J. (2011). Drug-specific risk of non-tuberculosis opportunistic infections in patients receiving anti-TNF therapy reported to the 3-year prospective French RATIO registry. Ann. Rheum. Dis..

[43] Sitapati A.M., Kao C.L., Cachay E.R., Masoumi H., Wallis R.S., Mathews W.C. (2010). Treatment of HIV-related inflammatory cerebral cryptococcoma with adalimumab. Clin. Infect. Dis..

[44] Scemla A., Gerber S., Duquesne A., Parize P., Martinez F., Anglicheau D., Snanoudj R., Zuber M., Bougnoux M.E., Legendre C. (2015). Dramatic improvement of severe cryptococcosis-induced immune reconstitution syndrome with adalimumab in a renal transplant recipient. Am. J. Transplant..

[45] Kuang D.M., Zhao Q., Peng C., Xu J., Zhang J.P., Wu C., Zheng L. (2009). Activated monocytes in peritumoral stroma of hepatocellular carcinoma foster immune privilege and disease progression through PD-L1. J. Exp. Med..

[46] Gordon S., Taylor P.R. (2005). Monocyte and macrophage heterogeneity. Nat. Rev. Immunol..

[47] Meya D.B., Okurut S., Zziwa G., Rolfes M.A., Melander K., Cose S., Joloba M., Naluyima P., Palmer B.E., Kambugu A. (2014). Cellular Immune Activation in Cerebrospinal Fluid from Ugandans with Cryptococcal Meningitis and Immune Reconstitution Inflammatory Syndrome. J. Infect. Dis..

[48] Chang C.C., Omarjee S., Lim A., Spelman T., Gosnell B.I., Carr W.H., Elliott J.H., Moosa M.Y., Ndung’u T., French M.A. (2013). Chemokine levels and chemokine receptor expression in the blood and the cerebrospinal fluid of HIV-infected patients with cryptococcal meningitis and cryptococcosis-associated immune reconstitution inflammatory syndrome. J. Infect. Dis..

[49] Jarvis J.N., Meintjes G., Bicanic T., Buffa V., Hogan L., Mo S., Tomlinson G., Kropf P., Noursadeghi M., Harrison T.S. (2015). Cerebrospinal Fluid Cytokine Profiles Predict Risk of Early Mortality and Immune Reconstitution Inflammatory Syndrome in HIV-Associated Cryptococcal Meningitis. PLoS Pathog..

[50] Scriven J.E., Rhein J., Hullsiek K.H., von Hohenberg M., Linder G., Rolfes M.A., Williams D.A., Taseera K., Meya D.B., Meintjes G. (2015). Early ART After Cryptococcal Meningitis Is Associated With Cerebrospinal Fluid Pleocytosis and Macrophage Activation in a Multisite Randomized Trial. J. Infect. Dis..

