# Supplementary material for: Monocyte Phenotype and IFN-γ-Inducible Cytokine Responses Are Associated with Cryptococcal Immune Reconstitution Inflammatory Syndrome
Source: J Fungi (Basel). 2017 Jun 2;3(2):28. doi: 10.3390/jof3020028 (PMC5715914; doi:10.3390/jof3020028)
Supplement: Supplementary file 1 [file jof-03-00028-s001.docx]

The following are available online at www.mdpi.com/2309-608X/3/2/28/s1.

Figure S1. Proportions of CD4^+^ and CD8^+^ T cells at Cryptococcal Diagnosis versus risk of future Cryptococcal-IRIS

Figure S2. LPS-induced Cytokine responses at Cryptococcal Diagnosis among Participants with and without Cryptococcal-IRIS.,

Figure S3. Comparison of IL-6 and TNF-α expression by monocyte populations at time of CM-IRIS vs time-matched CM Controls.

Figure S4. Comparison of IL-6 expression by monocyte populations for different Experimental Conditions

Appendix A


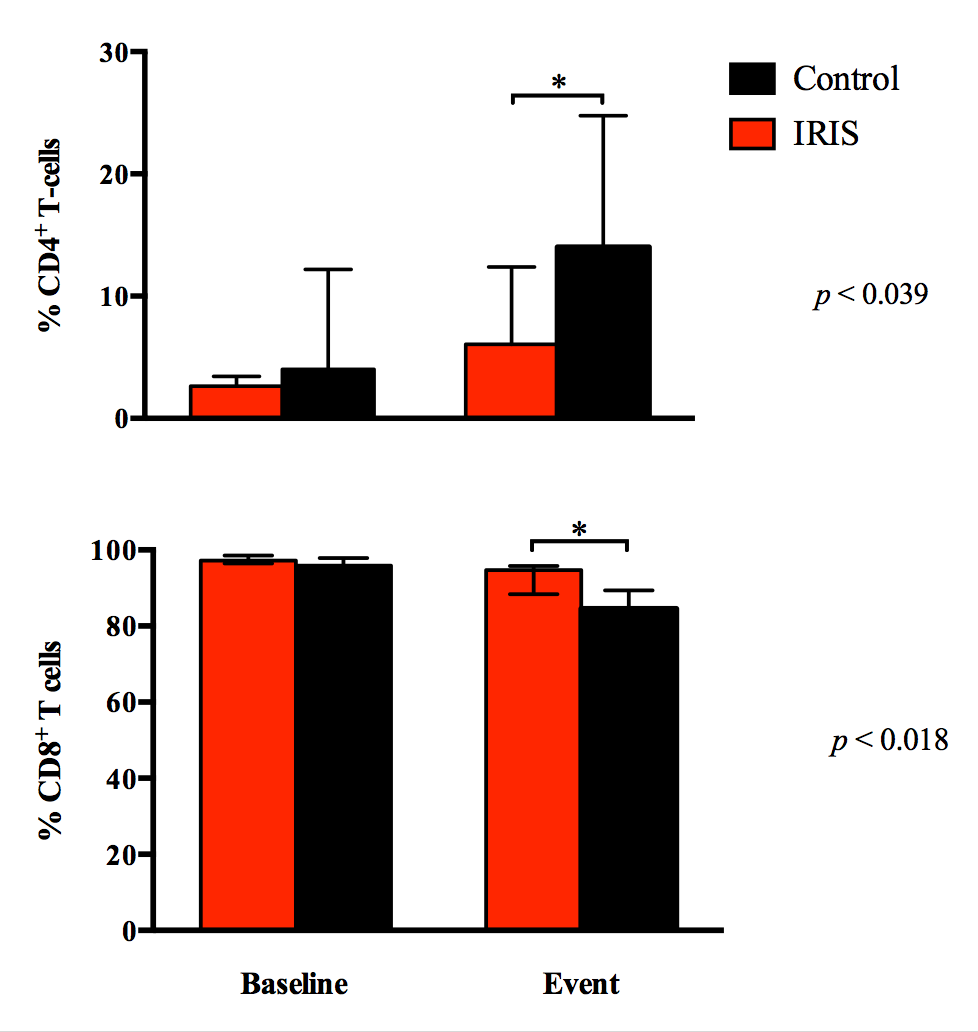


**Figure S1.** The frequencies of %CD4 T cells and %CD8 T cells were similar at Control-Baseline and IRIS-Baseline (left). Conversely, at IRIS-Event we observed a reconstituted lower frequency of %CD4 T cells and higher frequency of %CD8 T cells than Control-Event (right). Median frequencies were compared using the Mann-Whitney test**.**

**Figure S2.** IL-6 expression from LPS-stimulated cells was compared to IL-6 expression from unstimulated cells at IRIS-Baseline and Control-Baseline. *p*-values were determined by Mann-Whitney rank sum test. At IRIS-Baseline, we observed a trend to elevated intracellular IL-6 production following LPS stimulation, while at Control-Baseline, we found a significantly elevated intracellular expression of IL-6 following LPS-stimulation.

**Figure S3.** Comparison of IL-6 and TNF-α intracellular expression in indicated monocyte populations at IRIS-Event and Control-Event. Activated (PD-L1^+^CD25^+^) total monocytes (**Panel** **A**); Activated (PD-L1^+^CD25^+^) Classical monocytes (**Panel** **B);** Activated (PD-L1^+^CD25^+^) Intermediate monocytes (**Panel** **C)**. At IRIS-Event, IL-6 and TNF-α production was higher in the classical and intermediate monocyte subsets in both unstimulated cells and interferon-γ stimulated cells.


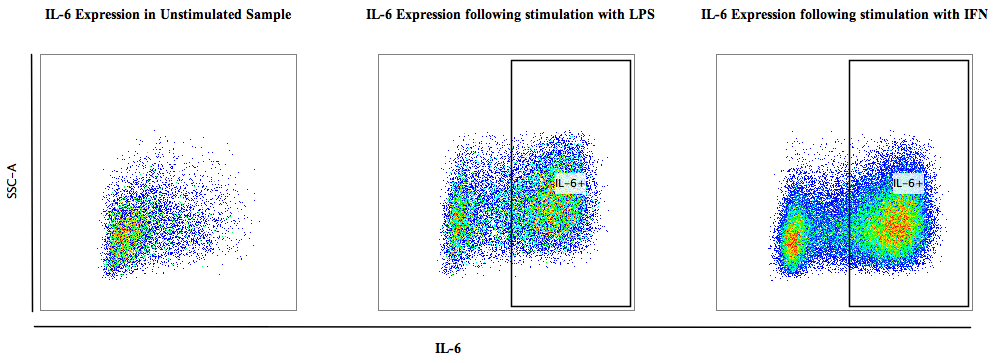


**Figure S4.** Comparison of IL-6 expression by monocytes from a representative sample under different experimental conditions.
